# Evaluation for Granulomatous Inflammation on Fine Needle Aspiration Cytology Using Special Stains

**DOI:** 10.4061/2011/851524

**Published:** 2011-06-30

**Authors:** Muhammad Mudassar Majeed, Mulazim Hussain Bukhari

**Affiliations:** Department of Pathology, King Edward Medical University, 26 MOF, GOR-3, Shahdman-Lahore 54000, Pakistan

## Abstract

*Background*. Tuberculosis is the commonest infectious disease in the developing world. Many diagnostic tests are devised for its detection including direct smear examination. This study was designed to determine the frequency of cases positive for AFB and positive for fungus in patients diagnosed to have granulomatous inflammation on Fine Needle Aspiration Cytology using special stains. *Materials and Methods*. A descriptive cross-sectional survey was done on 100 cases of granulomatous inflammation consistent with tuberculosis diagnosed on fine needle aspiration cytology at the Department of Pathology, King Edward Medical University, Lahore. After reporting granulomatous inflammation on Hematoxylin & Eosin staining of aspirates from FNAC, some unstained slides were subjected to special stains, like ZN, GMS, and PAS. Cases positive for AFB on ZN stain and fungus on GMS/PAS were noted down along with their frequency and percentages. *Results*. Forty-four cases (44%) of AFB positive smears were reported in granulomatous inflammation while only 5% cases of fungus were reported down. Cervical lymph nodes were the most commonly involved site (87%), and females were affected more (62%) than males. Most cases of AFB-positive smears were associated with caseation necrosis (93%). *Conclusion*. Special stains should be done on all granulomatous inflammation cases seen on FNAC for confirmation of TB and ruling out other infectious causes.

## 1. Introduction

Tuberculosis is playing havoc throughout the world, and this is especially true for the developing countries. Every year 8 million new cases are seen and 2 million deaths occur because of Tuberculosis [1]. In Pakistan, the estimated incidence of Tuberculosis is 181/100000 [2]. Tuberculosis (TB) carries a high risk of morbidity and mortality. TB has widespread involvement and rarely any tissue or organ is not involved by it. Most common is the pulmonary involvement [3] which has caused numerous deaths in the past. It can also involve the appendix [4], small and large intestine [5], skin [6], soft tissues, lymph nodes [7], genitourinary tract [8], and brain [9]. The dilemma does not end here and many other unusual organs are also involved [10].

The histology of TB is a characteristic showing granuloma formation by epithelioid histiocytes and Langhan's type Giant cells with or without caseation necrosis. This pattern is also preserved somehow in cytology specimens [11]. Infectious causes most notably presenting with granulomatous inflammation is Mycobacterium Tuberculosis with a reported frequency of 59.4% [11] and fungal causes [12, 13] with a reported frequency of 20.4% [14]. Other common causes include Sarcoidosis [15], Wegener's granulomatosis [16], Actinomycosis [17], Crohn's diseases [18], Histoplasmosis [19], foreign body, and Langerhans cell histiocytosis [20].

Pertaining to a broad differential diagnosis, the diagnosis of tuberculosis remains a challenge. History and clinical examination are always very helpful. Many diagnostic tests are in practice. Every test has its own sensitivity and specificity and limitations. The commonly performed tests include examination of sputum for Acid Fast Bacilli [21], Cultures for Mycobacterium tuberculosis [22], Fine Needle Aspiration Cytology (FNAC) [23], Biopsy, and PCR [24]. 

Fine Needle Aspiration Cytology is a minimally invasive and time-saving procedure, which helps in the diagnosis of number of diseases especially in palpable nodules of breast, lymph node disorders [25], thyroid [26], and palpable skin and subcutaneous nodules. It has become very popular nowadays among physicians and surgeons because of its benefits. In clinical practice, it helps them to reach a diagnosis or at least plan beforehand the proper management of the patient. As we have already discussed that granulomatous inflammation is not diagnostic of TB, many others causes must be ruled out before giving ATT. However, in the clinical scenario if a patient is diagnosed as granulomatous inflammation, then antituberculous treatment (ATT) is started at the first point in our setup. Statistically this behavior may be right but this is not in accordance with the reality. We come to encounter cases which have taken ATT for at least 9 months but still these symptoms persist. Reassessment is done, and later the patient is diagnosed as suffering from fungus, sarcoidosis, or some other granulomatous disease. Some special stains are very helpful in this regard, like Gomori Methenamine silver stains (GMS), Giemsa stain, Periodic acid Schiff (PAS), and Zeihl Neelson's stain (ZN stain) [27].

In the present study, granulomatous inflammation consistent with Tuberculosis diagnosed on FNAC will be analyzed using special stains like ZN (Ziehl Neelson's) and GMS (Gomori Methenamine Silver) stains. This will help to confirm tuberculosis in cases which will be positive for Acid Fast Bacilli on ZN staining. Positive GMS/PAS staining will confirm in the fungal causes of granulomatous inflammation including Mucormycosis, Blastomycosis, Cryptococcosis, and Candidiasis. There is a limitation of this study that not all causes of granulomatous inflammation can be ruled out since the ancillary investigations needed to diagnose them are not available in our setup.

The rationale of this study is that cases diagnosed wrongly as TB can turn out to be fungus and can be picked by GMS/PAS. These patients can thus be saved from long painful and harmful side effects of expensive ATT (Antituberculous therapy). This benefit alone is worth mentioning for the usefulness of this study, and this would be further reaffirmed by those who have experienced taking ATT for 9 months in their life without having TB. On ZN staining, the positive AFB cases would help the physicians to start treatment of TB, very confidently. Moreover, the work done to assess the frequency of different infectious agents in granulomatous inflammation especially fungal causes is very old, and this study would bridge a gap between newer studies done on this topic.

## 2. Material and Methods

### 2.1. Setting

The study was conducted at Pathology Department of King Edward Medical University and Mayo Hospital Lahore. The department receives 10,000 surgical specimens and 3000 cytology specimens including FNAC annually.

### 2.2. Duration

Six months.

### 2.3. Sample Size

Sample size of 100 cases was calculated with 95% confidence level, 8% margin of error, and taking expected percentage of positive cases of fungus on GMS/PAS that is, 20.4% in diagnosed cases of granulomatous inflammation.

### 2.4. Sampling Technique

Nonprobability purposive sampling.

### 2.5. Inclusion Criteria

1. Cases diagnosed on FNAC as granulomatous inflammation consistent with tuberculosis as per operational definitions.
2. Cases in which FNAC was done on Lymph node, skin swellings, subcutaneous swellings, and Lung masses

### 2.6. Exclusion Criteria

1. Pyogenic inflammation seen on microscopy as extensive neutrophilic infiltration.
2. Acellular smears/smears with crushed morphology or poorly stained slides will be excluded.
3. Previously diagnosed cases and cases already getting ATT.

### 2.7. Study Design

Descriptive cross-sectional survey.

### 2.8. Operational Definitions

#### 2.8.1. Granulomatous Inflammation

It is defined on cytology as aggregates of epitheloid cells forming a granuloma with or without necrosis. Sometimes multinucleated giant cells are also seen.

#### 2.8.2. Positive for AFB

On ZN staining the acid fast bacilli would be labeled when we find pink, beaded, and rod-shaped organisms after comparing with control samples.

#### 2.8.3. Positive for Fungus

On GMS staining, presence of black colored septated or nonseptated hyphae (depending upon the species of Fungus) or spores against a greenish background would be labeled as positive for fungus. On PAS stain, presence of red- or purple-colored septated or nonseptated hyphae or spores would be labeled as positive for fungus.

### 2.9. Data Collection Procedure

Patients fulfilling inclusion and exclusion criterion were selected from Fine needle aspiration cytology specimens received during the study period. After informed consent of patients and noting down the demographic data, the *hematoxylin* and Eosin staining was done. Two extra unstained slides were smeared from aspiration material. One slide was stained by GMS stain. Some cases were stained with Periodic Acid Schiff stain (PAS). Steps of PAS staining are as follows: similarly 2nd unstained slide was stained with Ziehl Nelson's stain. Commercially available positive and negative controls of ZN and GMS were used to compare and measure the consistency of staining technique. These smears were examined under the light microscope by a histopathology's. The findings of Hematoxylin and eosin staining were categorized as epitheloid granuloma with necrosis and epitheloid granuloma without necrosis. The finding of ZN staining was labeled as positive for AFB or negative for AFB. The finding of GMS was recorded as positive for fungus or negative for fungus.

### 2.10. Data Analysis

Data was analyzed by SPSS version 10. Age of patient was presented as mean and standard deviation. Gender, positive cases of AFB, and positive cases of fungus were presented as frequency and percentages.

## 3. Results

**Figure 1:**
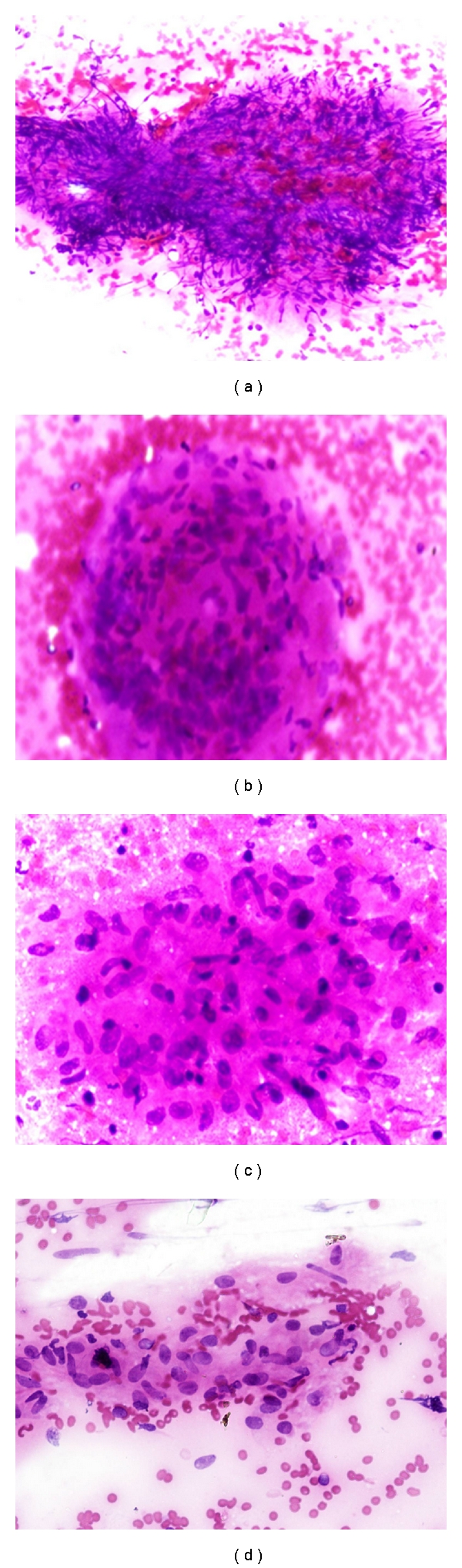
(a) Photomicrograph showing degenerated granulomas with caseation necrosis (H & E stain, 100x), (b) Granulomatous inflammation on FNAC (H & E stain, 200x) giant cells, (c) Granulomas comprising of epithelioid histiocytes with caseation necrosis (H & E stain, 200x), (d) Aggregates of pale staining epithelioid histiocytes (H & E, 400x).

**Figure 2:**
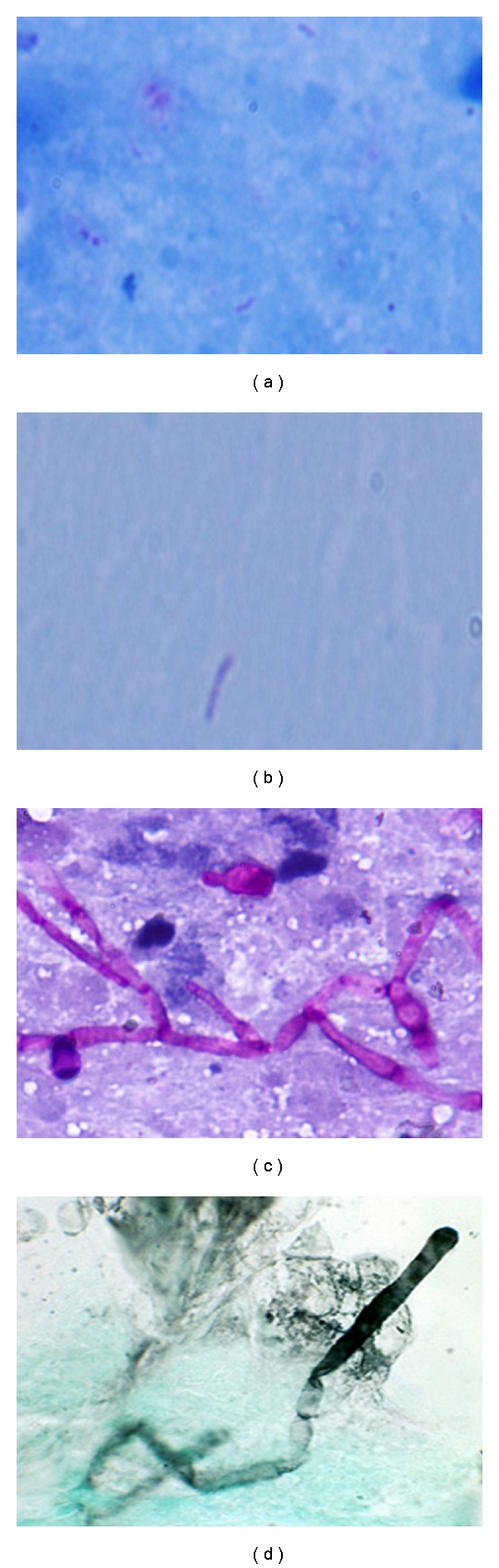
Photomicrographs in (a) Acid Fast Bacilli (Ziehl Nelson's stain, 400x), (b) Pink beaded rod against a blue background (ZN stain, 1000x), (c) Septated fungal hyphae and budding spore (at the top of photomicrograph), (PAS, 400x), (d) Black fungal hyphae against greenish background (GMS, 400x).

One hundred patients of granulomatous inflammation diagnosed on FNAC were taken. Granulomas were described as comprising of pale staining epithelioid cells which were round to oval to spindle against an eosinophilic background (Figure 1). Few degenerated epithelioid histiocytes were also seen in long-standing mycobacterial infection with caseation necrosis in the background (Figure 2). On Ziehl Neelson's staining, mycobacterium tuberculosis appeared as red/pink beaded rod-shaped bacteria against a blue background (Figures 2(a) and 2(b)). On PAS staining, fungus appears as purple hyphae which were segmented or nonsegmented depending on the species. Few spore forms with budding were also seen (Figure 2). On GMS stain, fungal hyphen appeared as black-colored forms which showed segmentation and some were nonsegmented (Figure 2).

In this study, 78% patients were below 30 years of age (Table 1). Mean age was 25.14 with standard deviation of 12.745. Females were affected more (68%) than males (Table 2). 44 out of 100 patients of granulomatous inflammation are positive for AFB (Table 3). There was an association between AFB positivity and caseation necrosis. We have found 41 out of total 44 AFB positive cases (93%) with caseation necrosis (Table 6), while 60% cases of fungus were related to caseation (Table 7). No definite relationship was seen between AFB and giant cells since 19 out of total 44 AFB positive cases were seen with caseation while rest 57% were without giant cells. Another finding was the involvement of specific lymph nodes regions. In 87% of cases, the most commonly involved group of lymph nodes was cervical lymph node (combining cervical and supraclavicular lymph nodes). If per auricular lymph nodes were included, then in 93% of cases the head and neck was the primary site of TB involvement (Table 5). 

## 4. Discussion

Accurate and timely diagnosis together with effective TB treatment is the mainstay of TB care and control. A confirmed diagnosis of TB can only be given on isolating the M. tuberculosis or finding specific DNA sequence of the bacteria in aspirates. In the resource-poor countries, however, these tests are not within the reach of every individual. In these countries, cost-effective techniques for example, sputum smear microscopy and morphological features are the corner stone of TB diagnosis. In cases of extra pulmonary tuberculosis, fine needle aspiration cytology (FNAC) is a very useful and reliable test. In areas where tuberculosis is prevalent, diagnosis of TB can be made by seeing the morphological features. Granulomatous inflammation is the common histological presentation of tuberculosis. However, there are many other infectious and noninfectious causes which can lead to granulomatous inflammation. Second important infectious cause of granulomatous inflammation is fungus. In the present study, we tried to differentiate between granulomatous inflammation caused by TB and fungus, by using special stains.

Blind FNAC can approach safely the superficial lesions, including lymph nodes, skin, and soft tissue nodules. In our study, 98 (98%) cases were from lymph nodes. Many studies have diagnosed TB by aspiration form lymph nodes [7, 11, 26, 28, 29]. Cervical lymph node was the most common site of involvement in studies followed by axillary lymph nodes [11, 21]. Our study was also consistent with above studies in terms of cervical lymph node involvement (87%) as the most common anatomic site of granulomatous inflammation. Periauricular lymph nodes were involved in 6% cases in our study and was the second most commonly involved. Female gender was a slightly more affected (62%) in current study and was in concordance with other studies [26]. However, there was slight male predominance in a study of Bezabih et al. [11]. Out of 100, 47% patients in this study were of 20 years or below and 62% were below 30. This finding was in accordance with Bezabih et al. in which 69% were below 30. Based on the facts, it can be inferred that tuberculosis was more commonly seen in young population [11]. 

One case of granulomatous inflammation was from skin (Table 5). Few studies from India have also discussed this aspect [30]. Numerous morphological variations in the granulomatous inflammation are seen. There were 69% cases with necrosis. The rest (31%) of cases were granulomatous inflammation without necrosis. The various morphological presentations of TB have been published locally [7]. International data also supports this variation and studies tried to correlate morphological findings with the AFB staining [11, 31]. 

The Acid Fast Bacilli positivity was labeled after finding red or pink rod-shaped bacteria with beaded appearance (Figures 2(a) and 2(b)). Regarding AFB positivity variable, results were seen and frequency ranges from 10% to 70% [26, 27, 30, 31]. 

In current study, out of 100 cases, 44 cases were positive for AFB (44%). This was in concordance with the international data of a large-scale study of 328 cases, out of which 152 cases (46.4%) were positive for AFB [21]. Similarly, our findings agree with Lau et al. who report 47% sensitivity for tuberculous abscess cases [26] and with Das et al. showing overall 45.8% rate of AFB positivity [32]. A study conducted in India shows an overall 27% AFB positivity [31], and the reason for this low AFB sensitivity was given: studies with higher AFB have adult subjects, in whom open tuberculosis and necrotic lesion were far more common. Examples of low yield of AFB were also due to treatment with antituberculosis drugs and presence of very few bacilli in the lymph node [31]. Some studies report very high frequency of AFB positivity. Bezabih et al. reported 59.4% of overall AFB positivity [30], and Vignesh et al. reported 53.3% sensitivity for single AFB smear [27].

In regions where TB is very common, the morphological findings of granulomatous inflammation is consistent with tuberculosis [30, 31]. Pakistan is also included among these countries along with India, Ethiopia, and other African countries. Since epithelioid granulomas, caseation necrosis, giant cells, and AFB positivity are specific for TB, so in these countries excision biopsy can be avoided and antituberculous treatment can be given straightaway [26]. Excision is not free of complication and is expensive and time consuming, thus it can delay the treatment. Above findings conclude that FNAC with special stains can solely help the physician to start the treatment. 

There was an interesting finding in our study. AFB positivity was notablely and more commonly found in granulomatous inflammation with caseation necrosis. 41 out of 44 AFB positive cases associated with caseation necrosis (93%), in current study. This finding is consistently seen in previous studies [11, 21, 31]. Otherwise in some studies, it is claimed that instead of granulomatous inflammation, if only necrosis or abscess formation is seen, the AFB-positivity increases [26]. Dua et al. even documented 100% of AFB positive cases in this scenario [31]. Since in the inclusion criterion of our study we only selected cases with granulomatous inflammation with or without necrosis, but not cases only with necrosis, this aspect cannot be discussed in this study. Most of studies improved the technique of finding AFB by using fluorescence microscopy. They claimed at least 10% improvement in sensitivity and sensitivity if fluorescence microscopy is used as compared to direct smear examination [33, 34]. However, in resource-poor countries it would still take some time to gain wide acceptance. 

Another interesting finding was that an acid fast bacillus was usually found extracellularly. Usually areas of microscopic degeneration, within or at the periphery of the granulomas, were most the common location to find AFB [21]. The morphology of these bacilli was short and stumpy rods with red beaded appearance. These findings correlated with those given by Rajasekaran et al. [35] and Ahmad et al. [21]. For early lesions of tuberculous lymphadenopathy, there is no evidence that chemotherapy (ATT) plus excision is superior than chemotherapy alone [26]. Moreover, the excision biopsy in tuberculous lymph nodes is hazardous since it may cause sinus formation. Therefore, FNAC finding of granulomatous inflammation and detection of AFB would be very specific and help the physicians to start ATT confidently, immediately as it is cost effective and economical.

The special stains GMS and PAS were used to detect the fungus, since it may present with same morphology as TB [14, 17, 36]. In this study, we found 5% cases of fungus presenting with granulomatous inflammation. After extensive search of the literature, only one study was found in which 20.4% cases of fungus occurred among 245 subjects [14]. Yet many other studies discussed fungus as a cause of granulomatous inflammation and published them as case reports [14, 36–39]. But these studies did not mention frequency or percentage of positive case of fungus. In this regard, the present study would bridge a gap and may become a source of future reference for further studies in this aspect. The main benefit we gained from this study was that these patients were diagnosed morphologically as “consistent with tuberculosis”. However, the results via special stains established that it can be caused by fungus and not only by mycobacterium tuberculosis. Added benefit is that these patients would be safe from harmful side effects of prolonged ATT treatment. They can get antifungal treatment, and the disease can be cured. In this study, we did not classify species of fungus on these special stains for it may not be accurate. For this purpose, fungal cultures should be performed.

**Recommendations**

1. Every case of granulomatous inflammation seen on aspiration cytology should be subjected to special stains like ZN, GMS/PAS. It would increase the diagnostic accuracy of this technique and help to differentiate between different infectious causes which can present with the same morphology.
2. When physicians are confronted with enlarged lymph nodes, the node may be punctured with a sterile disposable needle, and if cheesy material is aspirated then the physician can strongly consider tuberculous adenitis in areas where tuberculosis and immunodeficiency states are rampant and pathology services are lacking.
3. Patients who are not responding to empirical ATT should be considered for other causes of granulomatous inflammation other than TB, and proper workup should be done.

**Limitation of Study**

1. This study does not include comparison with histology and other microbiological detection methods like culture and PCR, because of cost and unavailability issues.
2. Our study did not comment on all the possible differential diagnosis of granulomatous inflammation, which requires sophisticated techniques and tertiary care laboratory services which are currently not available in our setup.

**Future Studies**

On the current issue, future studies should include comparison of direct smear microscopy of AFB with fluoroscopic evaluation. Moreover, the aspirate of FNAC should be subjected not only for special stains, but also for immunohistochemical stains, culture and PCR, and then compared for efficacy.

## 5. Conclusion

Fine needle aspiration cytology (FNAC) is very important investigation in the diagnosis of granulomatous inflammation. If it is supplemented with special stains like ZN, GMS, and PAS, it may help to differentiate between many infectious causes of granulomatous inflammation.

## Figures and Tables

**Table 1:** Distribution of age of patients.

Age of patient (years)	Granulomatous inflammation	% of granulomatous inflammation
1–10	3	3
11–20	44	44
21–30	31	31
31–40	11	11
41–50	8	8
51–60	2	2
61–70	0	0
71–80	1	1

Total mean = 25.14 ± 12.75	100	100

**Table 2:** Distribution of gender of patients.

Gender	Frequency	Percent (%)
Female	62	62
Male	38	38

Total	100	100

**Table 3:** Frequency of positive smears of acid fast bacilli.

Acid fast bacilli	Frequency	Percent
Positive for AFB	44	44
Negative for AFB	56	56

Total	100	100

**Table 4:** Frequency of fungus and granulomatous inflammation.

Fungus	Frequency	Percent
Negative for fungus	95	95
Positive for fungus	05	05

Total	100	100

**Table 5:** Distribution lesions according to site of FNAC.

Site of FNAC	Frequency	Percent	Valid percent	Cumulative percent
Cervical lymph node	72	72.0	72.0	72.0
Peri-auricular lymph nodes	6	6.0	6.0	78.0
Supraclavicular lymph node	15	15.0	15.0	93.0
Axillary lymph node	3	3.0	3.0	96.0
Inguinal lymph node	2	2.0	2.0	98.0
Skin or subcutaneous lesion	1	1.0	1.0	99.0
Other sites	1	1.0	1.0	100.0

Total	100	100.0	100.0	

**Table 6:** Relationship of acid fast bacilli with caseation necrosis.

	Acid fast bacilli		Total
	negative for AFB	positive for AFB	
Caseation necrosis			
Not present	28	3	31
Present	28	41	69

Total	56	44	100

**Table 7:** Frequency of caseation necrosis with fungus.

	Fungus		Total
	negative for fungus	positive for fungus	
Caseation necrosis			
Not present	29	2	31
Present	66	3	69

Total	95	5	100

**Table 8:** Frequency of acid fast bacilli with giant cells.

	Acid fast bacilli		Total
	negative for AFB	positive for AFB	
Giant cells			
Not present	50	25	75
Present	6	19	25

Total	56	44	100
